# A model based on C-TIRADS combined with SWE for predicting Bethesda I thyroid nodules

**DOI:** 10.3389/fonc.2024.1421088

**Published:** 2024-08-30

**Authors:** An Wei, Yu-Long Tang, Shi-Chu Tang, Xian-Ya Zhang, Jia-Yu Ren, Long Shi, Xin-Wu Cui, Chao-Xue Zhang

**Affiliations:** ^1^ Department of Ultrasound, The First Affiliated Hospital of Anhui Medical University, Hefei, China; ^2^ Department of Ultrasound, Hunan Provincial People’s Hospital/The First Affiliated Hospital of Hunan Normal University, Changsha, China; ^3^ Department of Thyroid Surgery, The Second Xiangya Hospital of Central South University, Changsha, China; ^4^ Department of Medical Ultrasound, Hunan Cancer Hospital/The Affiliated Cancer Hospital of Xiangya School of Medicine, Central South University, Changsha, China; ^5^ Department of Medical Ultrasound, Tongji Hospital, Tongji Medical College, Huazhong University of Science and Technology, Wuhan, China; ^6^ Department of Medical Ultrasound, Jingmen People’s Hospital, Jingmen, China

**Keywords:** ultrasound, thyroid nodule, elastography, the Bethesda system for reporting thyroid cytology, Chinese thyroid imaging reporting and data systems

## Abstract

**Objectives:**

This study aimed to explore the performance of a model based on Chinese Thyroid Imaging Reporting and Data Systems (C-TIRADS), clinical characteristics, and shear wave elastography (SWE) for the prediction of Bethesda I thyroid nodules before fine needle aspiration (FNA).

**Materials and methods:**

A total of 267 thyroid nodules from 267 patients were enrolled. Ultrasound and SWE were performed for all nodules before FNA. The nodules were scored according to the 2020 C-TIRADS, and the ultrasound and SWE characteristics of Bethesda I and non-I thyroid nodules were compared. The independent predictors were determined by univariate analysis and multivariate logistic regression analysis. A predictive model was established based on independent predictors, and the sensitivity, specificity, and area under the curve (AUC) of the independent predictors were compared with that of the model.

**Results:**

Our study found that the maximum diameter of nodules that ranged from 15 to 20 mm, the C-TIRADS category <4C, and *E*_max_ <52.5 kPa were independent predictors for Bethesda I thyroid nodules. Based on multiple logistic regression, a predictive model was established: Logit (p) = -3.491 + 1.630 × maximum diameter + 1.719 × C-TIRADS category + 1.046 × *E*_max_ (kPa). The AUC of the model was 0.769 (95% CI: 0.700–0.838), which was significantly higher than that of the independent predictors alone.

**Conclusion:**

We developed a predictive model for predicting Bethesda I thyroid nodules. It might be beneficial to the clinical optimization of FNA strategy in advance and to improve the accurate diagnostic rate of the first FNA, reducing repeated FNA.

## 1 Introduction

The global prevalence of thyroid nodules has ranged from 19% to 76% (1). Malignant nodules account for 10% to 15% of these and require clinical intervention (2, 3). The specificity of high-resolution ultrasound in the diagnosis of benign and malignant thyroid nodules is above 90%, which is of great value for the accurate differentiation of malignant thyroid nodules, but the sensitivity is only 26% to 87% (3–6).

To effectively identify and standardize the management of thyroid nodules, several Thyroid Imaging Reporting and Data Systems (TIRADS) have been proposed (7–10). The Chinese Thyroid Imaging Recording and Data System (C-TIRADS), introduced in 2020, is considered to have high accuracy and the lowest rate of unnecessary biopsies (7, 11, 12). In recent years, shear wave elastography (SWE) has received extensive attention in the differential diagnosis of thyroid nodules due to its ability to accurately quantify tissue stiffness (13–15). Two-dimensional ultrasound combined with SWE can further improve the diagnostic performance of thyroid nodules.

Fine needle aspiration (FNA) cytology is internationally recognized as a reliable and cost-effective method to identify thyroid nodules (16, 17). In 2010, the National Cancer Institute (NCI) established The Bethesda System for Reporting Thyroid Cytopathology (TBSRTC) to standardize terminology and promote communication. TBSRTC classifies FNA results into six categories: Bethesda I to Bethesda VI (18). Bethesda I thyroid nodules account for 2%–20%, and the risk of malignancy is up to 20% (19). Due to insufficient cell count or poor quality of specimens, Bethesda I thyroid nodules often cannot be diagnosed definitely. International guidelines recommend repeat FNA for Bethesda I thyroid nodules with suspicious ultrasound malignant signs (7, 18–21).

Therefore, Bethesda I thyroid nodules require more than two FNA or even diagnostic surgery to confirm the diagnosis. If Bethesda I thyroid nodules could be predicted in advance, core needle biopsy, performed by experienced operators, and on-site evaluation could be used to improve the diagnostic yield at the first FNA to avoid the financial burden and physical and mental stress caused by repeated FNA (18, 22, 23).

Previous studies have analyzed the ultrasound characteristics of Bethesda I thyroid nodules (24). However, it is not clear whether the ultrasound characteristics of thyroid nodules could be used to predict Bethesda I thyroid nodules. The predictive performance is expected higher not only based on the ultrasound features but also on the 2020 C-TIRADS category and SWE.

This study aimed to investigate the predictive performance of a model based on ultrasound, C-TIRADS, clinical characteristics, and SWE for the pre-puncture evaluation of Bethesda category I in patients with thyroid nodules, which might be beneficial to the clinical optimization of FNA strategy in advance and to improve the accurate diagnostic rate of the first FNA, reducing the economic and time cost caused by repeated FNA (22, 25, 26).

## 2 Materials and methods

### 2.1 Patients’ selection and data collection

A total of 1,724 patients with thyroid nodules who underwent both ultrasound and FNA examinations from January 2021 to April 2023 were retrospectively analyzed. FNA diagnosis was based on TBSRTC criteria. This study was approved by the Medical Ethics Committee of our hospital for waiver of informed consent (2023 No.: LY-2023-89). The inclusion criteria were as follows: (1) solid or predominantly solid thyroid nodules (75%), (2) age ≥18 years old, (3) ultrasound and SWE examination were performed within 1 month before FNA, and (4) FNA and ultrasound showed the same nodule. The exclusion criteria were as follows: (1) ultrasound image quality could not meet the requirements, (2) lack of SWE data of nodules, and (3) previous treatment history such as puncture and ablation. The nodules were divided into Bethesda I and Bethesda non-I groups according to the cytological results.

The patient’s age, gender, and other data were collected through the Picture Archiving and Communication System (PACS) and medical record system.

### 2.2 Ultrasound examination

A Super Sonic Aixplorer system (Super Sonic Imagine, Aix en Provence, France) was utilized to perform ultrasound and SWE examinations, which was equipped with an L15-4 linear array transducer. Clear ultrasound images of the targeted thyroid nodule were first obtained. A detailed record of the characteristics of the thyroid nodule was made, including the location, size, orientation, margin, echogenicity, echotexture, echogenic foci, and posterior features. All nodules were classified according to the C-TIRADS criteria (shown in the **Supplementary Material**). The length, width, and height of the nodules were measured from the maximum longitudinal section and the maximum vertical cross-section of the nodule, respectively. The maximum diameter value was included in the study.

Subsequently, SWE was performed using the same transducer. The quantification box (Q-box) method was employed to evaluate the elastic characteristics of the nodules, and the longitudinal section was used for SWE imaging. The stiffness range of the color map was adjusted from blue to red (0–180 kPa). The region of interest encompassed the nodule and part of the surrounding normal tissue, which constituted an area approximately two to three times the size of the nodule itself. The patients were required to hold their breath temporarily during imaging. SWE was measured using the quantitative box (Q-box), which was placed in the hardest part of the nodule, excluding the calcified area and adjacent tissue (6, 27). The system automatically calculates the maximum elastic modulus (*E*_max_), which was measured five times and averaged for analysis.

To reduce measurement errors, ultrasound and SWE examinations of all nodules in this study were performed by the same radiologist with more than 15 years of experience in thyroid ultrasound. Another radiologist who was trained in C-TIRADS and had more than 10 years of experience in the diagnosis of thyroid disease classified all nodules by C-TIRADS criteria (shown in the **Supplementary Material**) (7).

### 2.3 Statistical analysis

SPSS 26.0 and R 4.3.0 were used for statistical analysis. Kappa analysis or Fisher’s precision test was used to compare the clinical features, ultrasound features, and C-TIRADS categories of Bethesda I and non-I thyroid nodules. The threshold for statistical significance was *P* < 0.05. Statistically significant variables were obtained by univariate analysis. The receiver operating characteristic (ROC) curve was drawn to obtain the best cutoff value and area under the curve (AUC) to predict Bethesda I thyroid nodules. Statistically significant variables were added to multivariate logistic regression analysis to identify independent predictor variables, and a combined prediction model was constructed on this basis. The predictive efficacy of the independent predictors and the model was evaluated using the ROC method, and the AUC, sensitivity, and specificity of the independent predictors and the model were compared.

## 3 Results

### 3.1 Clinical data and ultrasound features between the Bethesda I and non-I thyroid nodules

**Figure 1:**
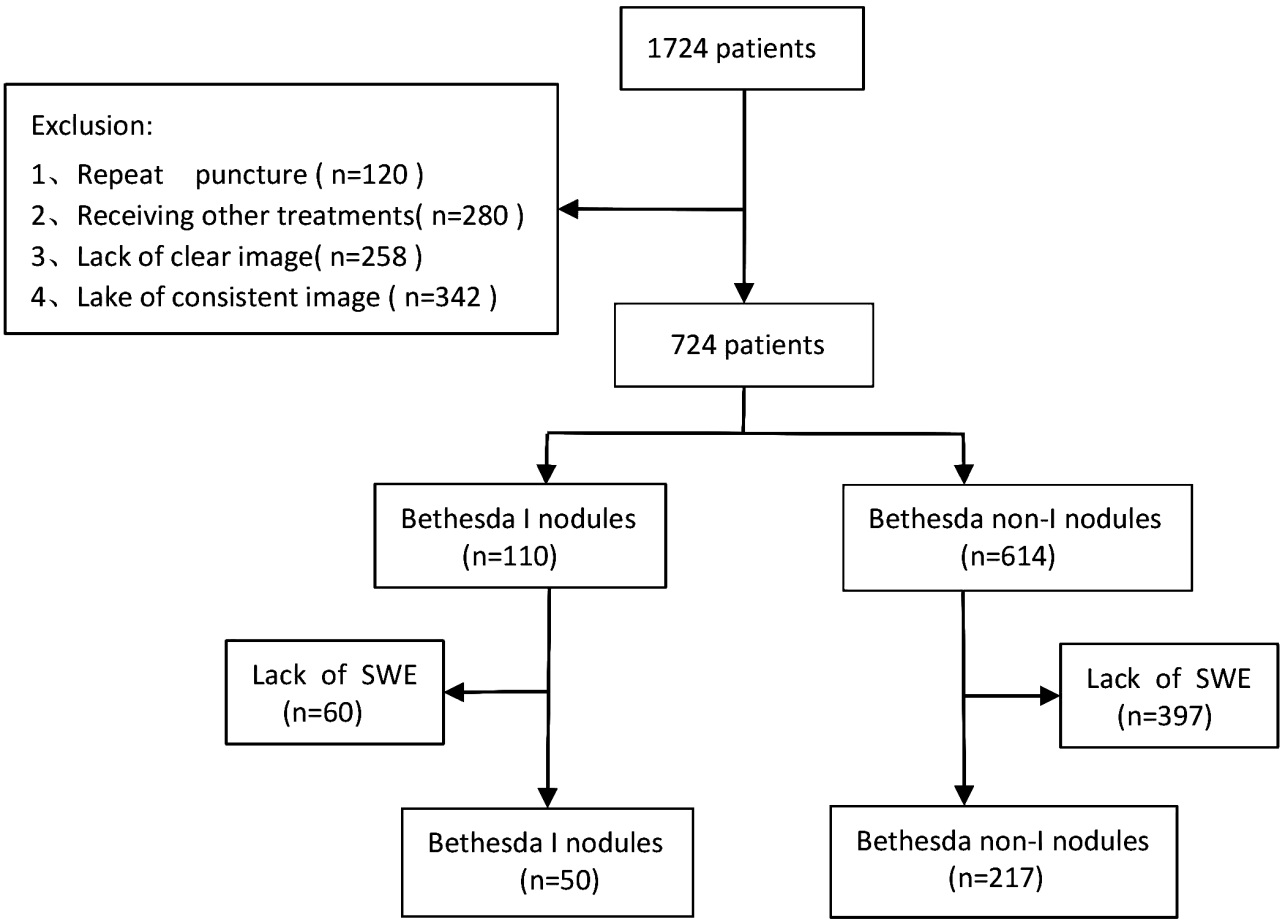
Patient selection flow chart of this study.

A total of 267 patients were finally enrolled, with an average age of (43 ± 12) years (208 female and 59 male) (**Figure 1**). There were 50 Bethesda I (18.73%) and 217 non-I thyroid nodules (81.27%). The clinical and ultrasound characteristics are summarized in **Table 1**. Only age was statistically different between the two groups. The other clinical and ultrasound features were not statistically significant.

**Table 1:** Conventional ultrasound features and clinical data features between the Bethesda I and Bethesda non-I group.

Variable	Number	Bethesda I group (*n* = 50)	Bethesda non-I group (*n* = 217)	*X* ^2^/*Z*/*t*	*P*
Sex	Male (*n* = 59)	12 (24.0%)	47 (21.7%)	0.129	0.719
	Female (*n* = 208)	38 (76.0%)	170 (78.3%)		
Echogenicity					
Hypoechoic	Y (*n* = 236)	42 (84.0%)	194 (89.4%)	1.155	0.282
	N (*n* = 31)	8 (16.0%)	23 (10.6%)		
Markedly hypoechoic	Y (*n* = 29)	5 (10.0%)	24 (11.1%)	0.030	0.862
	N (*n* = 237)	45 (90.0%)	193 (88.9%)		
Hyperechoic	Y (*n* = 2)	0 (0.0%)	2 (0.9%)	0.464	0.496
	N (*n* = 265)	50 (100%)	215 (99.1%)		
Isoechoic	Y (*n* = 12)	4 (8.0%)	8 (3.7%)	1.761	0.187
	N (*n* = 255)	46 (92%)	209 (96.3%)		
Echogenic foci					
Calcification	Y (*n* = 153)	31 (62.0%)	122 (56.2%)	0.555	0.456
	N (*n* = 114)	19 (38.0%)	95 (43.8%)		
Microcalcifications	Y (*n* = 123)	20 (40.0%)	103 (47.5%)	0.912	0.340
	N (*n* = 144)	30 (60.0%)	114 (52.5%)		
Margin					
Irregular margin	Y (*n* = 188)	37 (74.0%)	151 (69.6%)	2.586	0.108
	N (*n* = 79)	13 (26.0%)	66 (30.4%)		
Ill-defined margin	Y (*n* = 235)	43 (86.0%)	81.7 (37.6%)	0.237	0.627
	N (*n* = 32)	7 (14.0%)	25 (11.5%)		
Extrathyroidal Extension	Y (*n* = 155)	29 (58.0%)	126 (58.1%)	0.000	0.993
	N (*n* = 112)	21 (42.0%)	91 (41.9%)		
Orientation					
Taller-than-wide	Y (*n* = 161)	30 (60.0%)	131 (60.4%)	0.002	0.962
	N (*n* = 106)	20 (40.0%)	86 (39.6%)		
Regular form	Y (*n* = 45)	9 (18.0%)	36 (16.6%)	0.058	0.810
	N (*n* = 222)	41 (82.0%)	181 (83.4%)		
Homogeneous	Y (*n* = 7)	1 (2.0%)	6 (2.8%)	0.000	1.000
	N (*n* = 260)	49 (98.0%)	211 (97.2%)		
Location					
Inside	Y (*n* = 16)	0 (0%)	16 (7.4%)	2.722	0.099
	N (*n* = 251)	50 (100%)	201 (92.6%)		
Outside	Y (*n* = 22)	3 (6.0%)	19 (8.8%)	0.125	0.724
	N (*n* = 245)	47 (94.0%)	198 (91.2%)		
Deepside	Y (*n* = 47)	9 (18.0%)	38 (17.5%)	0.007	0.935
	N (*n* = 220)	41 (82%)	179 (82.5%)		
Shallow side	Y (*n* = 70)	14 (28.0%)	56 (25.8%)	0.101	0.751
	N (*n* = 197)	36 (72.0%)	161 (74.2%)		
Upper	Y (*n* = 26)	2 (4.0%)	24 (11.1%)	1.571	0.185
	N (*n* = 241)	48 (96.0%)	193 (88.9%)		
Lower	Y (*n* = 56)	14 (28.0%)	42 (19.4%)	1.832	0.177
	N (*n* = 211)	36 (72.0%)	175 (80.6 %)		
Position					
Left lobe	(*n* = 120)	23 (46.0%)	97 (80.8 %)	1.754	0.464
Right lobe	(*n* = 139)	24 (48.0%)	115 (53.0%)		
Isthmus	(*n* = 8)	3 (6.0%)	5 (2.3%)		
Age (years)	(*n* = 267)	46 ± 12	42 ± 12	-2.026	0.044*
≥60	(*n* = 21)	8 (16.0%)	13 (6.0%)	4.322	0.038*
<60	(*n* = 246)	42 (84.0%)	204 (94.0%)		

*P-value <0.05 was considered statistically significant.

### 3.2 Age features between the Bethesda I and non-I thyroid nodules

The age of patients with BethesdaIand non-I thyroid nodules exhibited a statistically significant difference (46 ± 12 years vs. 42 ± 12 years, *P* = 0.044). The ROC was drawn according to age to calculate the best cutoff value to distinguish two group nodules. The optimal cutoff value of age was 59 years old, which was close to 60 years old. The rate of Bethesda I thyroid nodules was higher in patients over 60 years old compared to those under 60 years old (16.0% vs. 6.0%, *P* = 0.038).

### 3.3 Maximum diameter features between the Bethesda I and non-I thyroid nodules

The maximum diameter of 267 nodules ranged from 3 to 50 mm (12.3 ± 9.4 mm). Nine nodules had a maximum diameter of less than 5 mm, all of which were classified as C-TIRADS 4C category. FNA was performed after obtaining the patient’s consent. One, five, and three nodules were classified as Bethesda VI, V, and III categories, respectively. Five cases were postoperative histopathology confirmed as papillary thyroid carcinoma. The maximum diameter features between the two groups are summarized in **Table 2**. The incidence of Bethesda I in thyroid nodules with a maximum diameter of 15–20 mm was significantly higher than that in nodules of other sizes (*P* = 0.014).

**Table 2:** Bethesda I rate of 267 nodules according to the maximum diameter.

Nodule size, mm	Bethesda I group, *n* (%)	*X* ^2^/*Z*/*t*	*P*
≤5 vs. >5		0.000	0.998
≤5, *n* = 24	5 (20.8)		
>5, *n* = 243	45 (18.5)		
≤10 vs. >10		1.145	0.285
≤10, *n* = 141	23 (16.3)		
>10, *n* = 126	27 (21.4)		
≤15 vs. >15		2.136	0.144
≤15, *n* = 198	33 (16.7)		
>15, *n* = 69	17 (24.6)		
≤20 vs. >20		0.414	0.520
≤20, *n* = 233	45 (19.3)		
>20, *n* = 34	5 (14.7)		
>5mm, ≤10 mm vs. others		0.216	0.269
>5mm, ≤10 mm, *n* = 117	18 (15.4)		
others, *n* = 150	32 (21.3)		
>10 mm, ≤15 mm vs. others		0.796	0.851
>10 mm, ≤15 mm, *n* = 57	10 (17.5)		
others, *n* = 210	40 (19.0)		
>15 mm, ≤20 mm vs. others		6.407	0.011*
>15 mm, ≤20 mm, *n* = 35	12 (34.3)		
others, *n* = 232	38 (16.4)		

*P-value <0.05 was considered statistically significant.

### 3.4 *E*_max_ between the Bethesda I and non-I thyroid nodules

The range of *E*_max_ of 267 nodules was 5–300 kPa, and the median value was 48.8 kPa (30–85 kPa). As shown in **Table 3**, there was a significant difference in *E*_max_ between Bethesda I and non-I thyroid nodules (35.95 vs. 56.6 kPa, *P* = 0.001). The lower the *E*_max_ value of thyroid nodules, the higher the incidence of Bethesda I thyroid nodules.

**Table 3:** Bethesda I rate of 267 nodules according to C-TIRADS and *E*_max_.

	Group		*X* ^2^	*P*
	Bethesda I (*n* = 50)	Bethesda non-I (*n* = 217)		
C-TIRADS			28.399	<0.001*
3 (*n* = 6)	3 (6.0%)	3 (1.4%)	2.122	0.145
4A (*n* = 42)	14 (28.0%)	28 (12.9%)	6.987	0.008*
4B (*n* = 84)	24 (48.0%)	60 (27.6%)	7.805	0.005*
4C (*n* = 84)	7 (14.0%)	77 (35.5%)	8.698	0.003*
5 (*n* = 51)	2 (4.0%)	49 (22.6%)	9.045	0.003*
<4C (*n* = 132)	41 (82.0%)	91 (41.9%)	26.095	<0.001*
≥4C (*n* = 135)	9 (18.0%)	126 (58.1%)		
*E*_max_ (kPa)	36.0 (25.3–54.3)	56.6 (31–90)	-3.346	0.001*
*E*_max_ <52.5 kPa	38 (76.0%)	101 (46.5%)	14.128	<0.001
≥52.5 kPa	12(24%)	116(53.3%)		

*P-value <0.05 was considered statistically significant.

C-TIRADS, Chinese Thyroid Imaging Reporting and Data Systems; *E*_max_, maximum elastic modulus.

An ROC was drawn according to *E*_max_ of nodules to calculate the best cutoff value to distinguish the two groups’ nodules. The optimal cutoff value of *E*_max_ was 52.5 kPa. Thyroid nodules with *E*_max_ values below 52.5 kPa were significantly more likely to be Bethesda I thyroid nodules than those with *E*_max_ values above 52.5 kPa (76.0% vs. 24.0%, *P* <0.001).

### 3.5 C-TIRADS categories between the Bethesda I and non-I thyroid nodules

As shown in **Table 3**, the constituent ratio of C-TIRADS categories of the two groups’ nodules was statistically different (*P* < 0.001). The majority of Bethesda I thyroid nodules were C-TIRADS 4A (28% vs. 12.9%, *P* = 0.008) and 4B category (48% vs. 27.6%, *P* = 0.005). However, Bethesda non-I thyroid nodules were mainly C-TIRADS 4C (35.5% vs. 14.0%, *P* = 0.003) and 5 categories (22.6% vs. 4.0%, *P* = 0.003). The difference was statistically significant. The ROC curve was drawn according to the C-TIRADS categories to calculate the best cutoff value to distinguish Bethesda I and non-I thyroid nodules. The optimal cutoff value was 4C. The risk of thyroid nodules with C-TIRADS 3 to 4B categories being Bethesda I thyroid nodules was higher than that of thyroid nodules with C-TIRADS 4C to 5 categories (*P* = 0.001).

### 3.6 Univariate and multivariate analysis on the predictors of Bethesda I thyroid nodules

The binary logistic regression analysis of clinical data, ultrasound, C-TIRADS category, and *E*_max_ value characteristics are summarized in **Table 4**. The maximum diameter of the nodules that ranges from 15 to 20 mm, C-TIRADS category <4C, and *E*_max_ value <52.5kPa were independent predictors for Bethesda I thyroid nodules. A combined predictive model of Bethesda I thyroid nodules was established based on multiple logistic regression: Logit (p) = -3.491 + 1.630 × maximum diameter + 1.719 × C-TIRADS category + 1.046 × *E*_max_ (kPa).

**Table 4:** Cutoff value and logistic regression analysis of risk factors predicting Bethesda I thyroid nodules.

Variable	Cutoff value	B	*P*	OR	95% CI for OR	
Age (years)	58.5	0.932	0.090	2.539	0.866	7.447
Maximum diameter (mm)	15-20	1.630	0.001*	5.106	1.982	13.151
C-TIRADS	<4C	1.719	<0.001*	5.577	2.406	12.929
*E*_max_ (kPa)	52.5	1.046	0.011*	2.848	1.277	6.349
Constant		-3.491	<0.001*	0.142		

*P-value <0.05 was considered statistically significant.

C-TIRADS, Chinese Thyroid Imaging Reporting and Data Systems; OR, odds ratio; *E*_max_, maximum elastic modulus.

### 3.7 Comparing the predictive performance of the combined predictive model and the independent predictors

**Figure 2:**
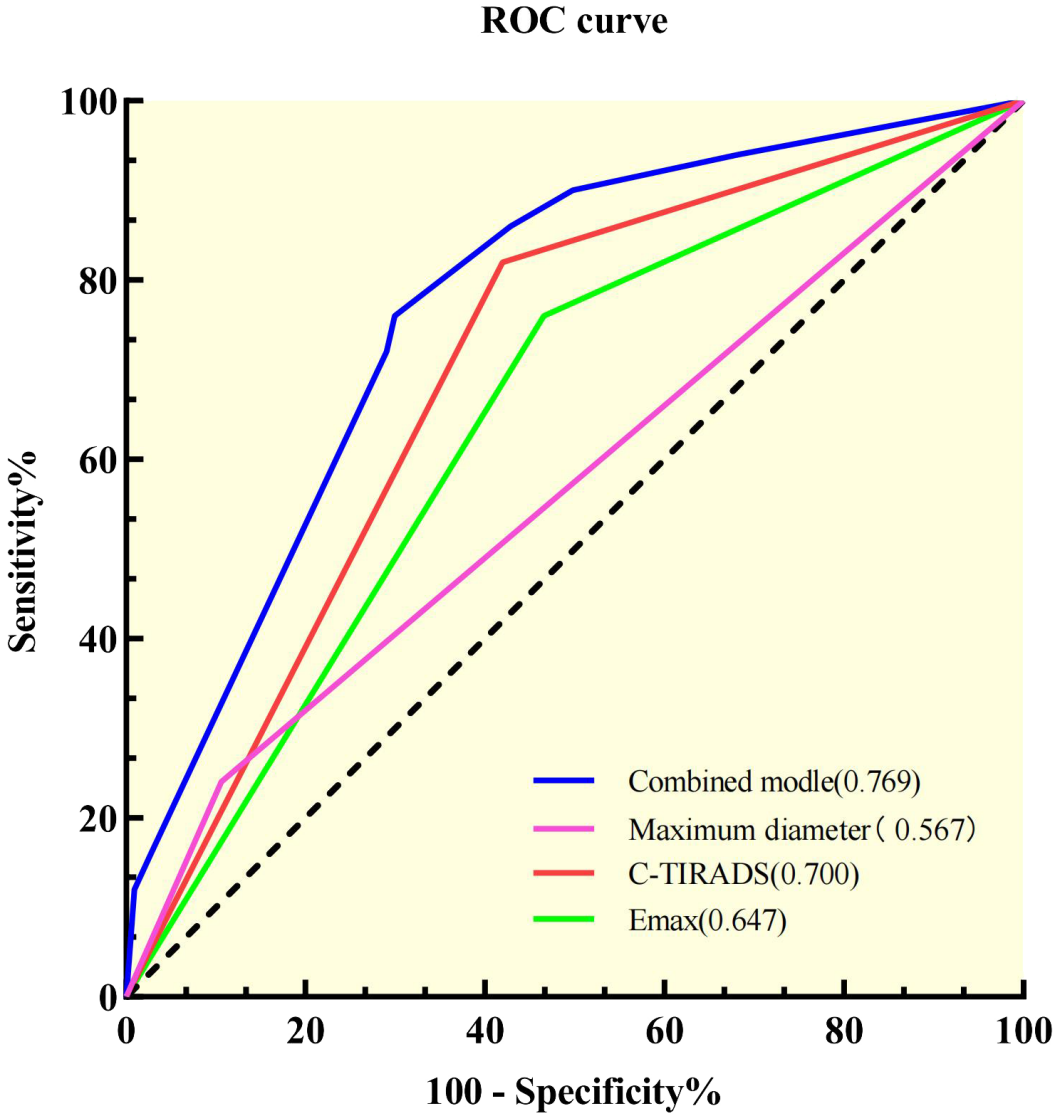
Receiver operating characteristic curves of the independent predictors and the combined model.

ROC analysis was performed on the model to assess Bethesda I thyroid nodules (**Figure 2**). The AUC of the model was 0.769 (95% CI: 0.700–0.838), which was significantly higher than that of C-TIRADS, *E*_max_, and other independent predictors alone (**Table 5**). The model also had the best sensitivity of 76% and a better specificity of 71.2% compared with the use of independent predictors alone.

**Table 5:** Prediction performance of combined predictive models and independent predictors.

	Sensitivity	Specificity	AUC	95% confidence interval	*P* ^a^
Maximum diameter (15–20 mm)	24%	83.6%	0.567	0.474–0.660	<0.001*
C-TIRADS (<4C)	18%	41.9%	0.700	0.625–0.776	0.001*
*E*_max_ (kPa)	75%	53.5%	0.647	0.566–0.728	<0.001*
Combined prediction model	76%	71.2%	0.769	0.700–0.838	

*P-value <0.05 was considered statistically significant.

a The *P*-value is the statistical difference between the AUC of the independent risk predictor and the AUC of the combined prediction model.

C-TIRADS, Chinese Thyroid Imaging Reporting and Data Systems; *E*_max_, maximum elastic modulus; AUC, area under the receiver operator characteristic curve.

## 4 Discussion

The study compared the basic clinical information and ultrasound imaging characteristics of Bethesda I and non-I thyroid nodules, revealing that the maximum diameter of the nodules that ranged from 15 to 20mm, C-TIRADS category <4C, and *E*_max_ <52.5 kPa were independent predictors to predict Bethesda I thyroid nodules. The AUC of a combined model based on maximum diameter, C-TIRADS category, and *E*_max_ in predicting Bethesda I thyroid nodules was 0.769, and the sensitivity and specificity were 76.0% and 71.2%, respectively. The combined model has better predictive performance than C-TIRADS or other independent predictors alone for Bethesda I thyroid nodules. We can optimize FNA strategies by predicting Bethesda I thyroid nodules in advance, such as selecting core needle biopsy, changing experienced operators, and on-site sample evaluation, to further improve the FNA diagnostic rate and reduce repeat FNA (21–23, 25).

A retrospective study found an effect of age on the incidence of Bethesda I thyroid nodules (28). The results of our study are consistent with it, and the optimal cutoff value was found to be 59 years. The risk of Bethesda I thyroid nodules in patients aged ≥60 years was 2.5 times that in patients aged <60 years, indicating a higher prevalence of Bethesda I thyroid nodules among elderly individuals.

Nodule size may influence the incidence of Bethesda I thyroid nodules. Dong et al. found that thyroid nodules with a diameter of more than 15 mm were more likely to be Bethesda I thyroid nodules (24). Our study found that thyroid nodules with a diameter range of 15 to 20 mm were five times more likely to be Bethesda I thyroid nodules than other-sized nodules. It is possible that larger nodules (>15 mm in diameter) are more likely to exhibit heterogeneity and may lead to false negative results if the sampling does not adequately cover the entire nodule at the time of FNA. As the size of the nodule increased, the risk of malignancy increased. Hypoechoic solid nodules larger than 15 mm in diameter are indications of FNA (29). For such high-risk nodules, guidelines recommend repeat biopsy, core needle biopsy, and even diagnostic surgery to avoid a leak of malignant nodules, which undoubtedly increases the personal and medical financial burden. Therefore, FNA is recommended for large thyroid nodules from multiple angles and sites while avoiding vascular-rich areas to ensure adequate tumor cell samples. FNA can be performed in contrast-enhanced ultrasound mode if necessary (30). In this study, we found that the incidence of Bethesda I thyroid nodules was not significantly different between nodules with a diameter less than 5 mm and those with larger sizes, which suggests that FNA can still yield satisfactory diagnostic results for nodules smaller than 5 mm.

There were no statistically significant differences in single ultrasound features between Bethesda I and non-I thyroid nodules. Previous studies have also suggested that a single ultrasound feature has limited predictive sensitivity (31). This problem could be solved by using TIRADS to comprehensively score the ultrasound features of nodules. C-TIRADS was proposed by the Chinese Society of Ultrasound Medicine in 2020 (7). Some studies found that C-TIRADS had the highest AUC (0.905 vs. 0.854, 0.805, and 0.863) and AUC (0.816 vs. 0.789, 0.773, 0.763, and 0.734), higher accuracy (84.71% vs. 82.11%, 81.64%, and 78.56%), and lower unnecessary biopsy rate (22.61% vs. 27.9% and 28.67%) (11, 12). Therefore, C-TIRADS was also used in this study to evaluate thyroid nodules as a whole, and it was observed that the incidence of Bethesda I thyroid nodules was lower in C-TIRADS categories 4C and 5 than in other categories. This might be because C-TIRADS has the highest positive predictive value among the various TIRADS guidelines, making its 4C and 5 categories more likely to represent malignant nodules (12).

The SWE technique is capable of visualizing the two-dimensional distribution of tissue stiffness. Tissue stiffness is quantified using Young’s modulus (kPa) and/or shear wave velocity (m/s), exhibiting high repeatability among different operators (6). Therefore, SWE can provide additional diagnostic information to distinguish between benign and malignant thyroid nodules. Several studies have demonstrated that among the various parameters of SWE, *E*_max_ can serve as the most reliable diagnostic index (6, 27, 32). In this study, it was observed that the *E*_max_ values differed between Bethesda I and non-I thyroid nodules. The optimal cutoff value to predict Bethesda I thyroid was 52.5 kPa, with a sensitivity of 75%. In other words, Bethesda I thyroid nodules were dominated by soft nodules, with 75% of the nodules having *E*_max_ values <52.5 kPa. This suggests that stiffer nodules might possess higher cell density, making it easier for FNA to obtain a cell specimen that meets the diagnostic criteria. The low incidence of Bethesda I thyroid nodules among C-TIRADS 4C and 5 categories of nodules found in this study is attributed to the high malignant probability and cell density associated with these two types of nodules, facilitating the acquisition of qualified cell samples during FNA.

**Figure 3:**
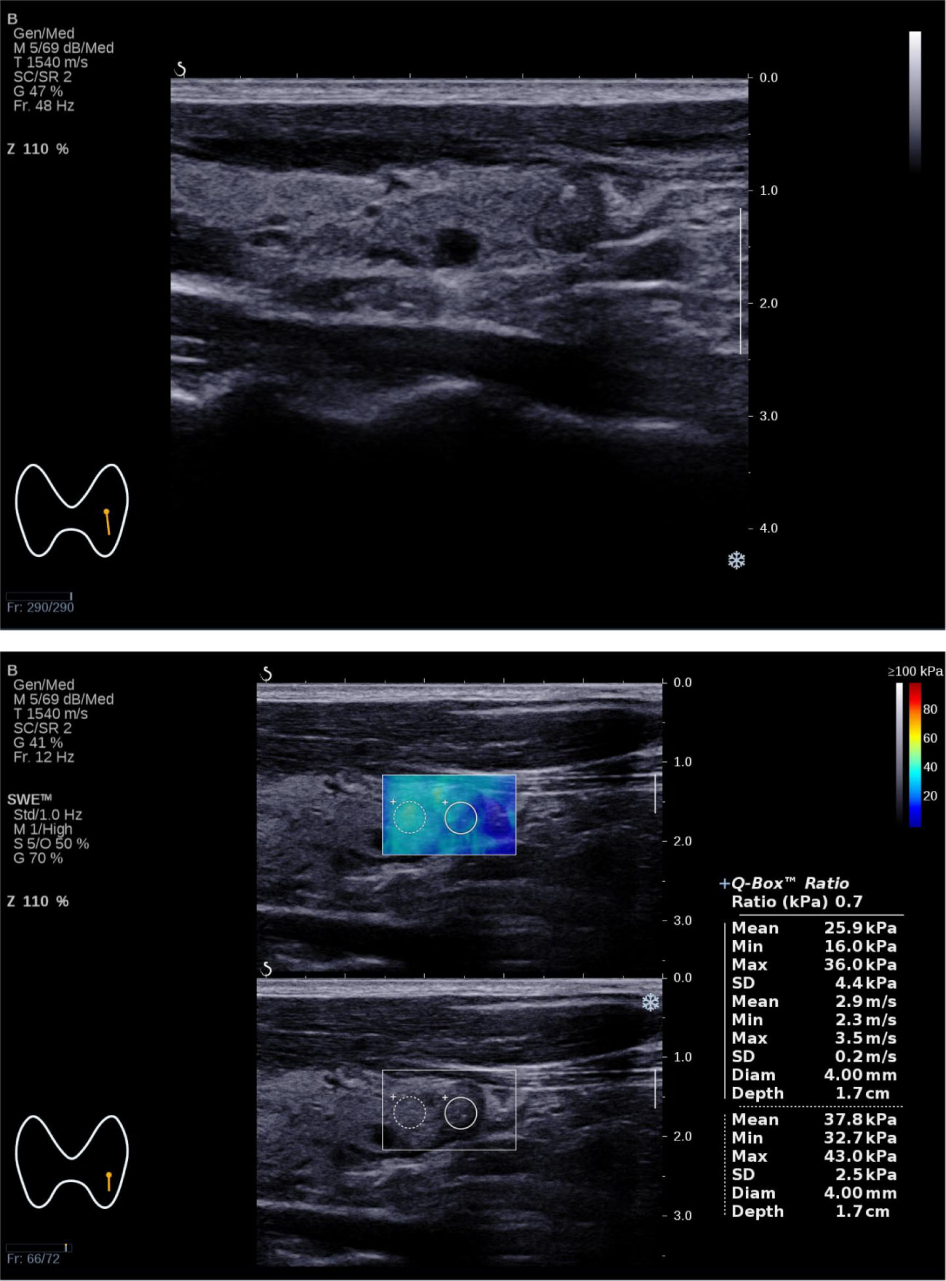
The Bethesda I thyroid nodules of a 31-year-old man was predicted with the combined model. A 31-year-old man with a 7×6×6 mm thyroid nodule in the left lobe. Conventional ultrasound showed a solid hypo-echoic, regular nodule taller than wider with coarse calcifications, which was categorized as C-TIRADS 4B. SWE measurement showed that *E*_max_ was 36.0 kPa. The predictive value calculated by the model was 0.712(>0.226), which might be considered a Bethesda I nodule. The FNA result was Bethesda I.

**Figure 4:**
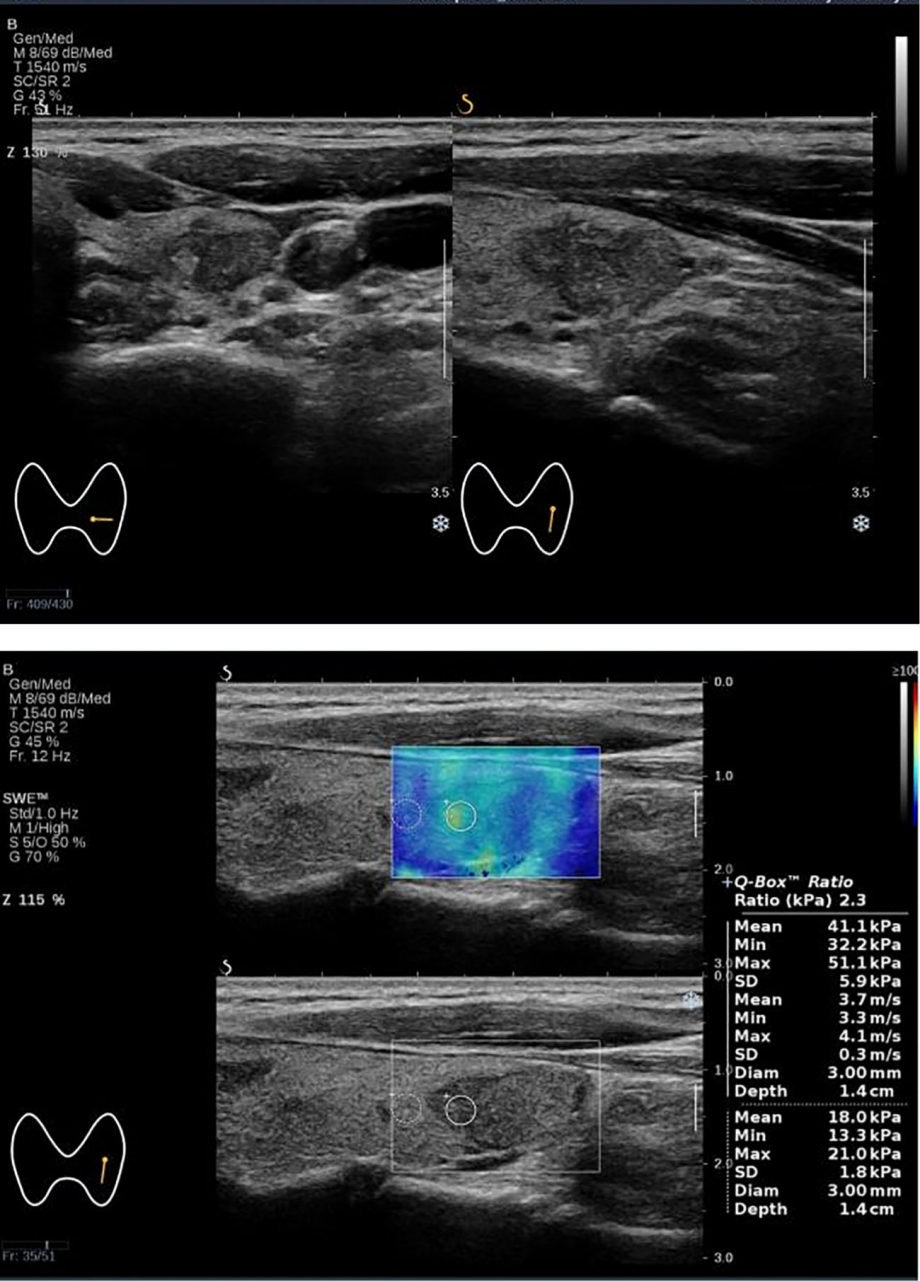
The Bethesda I thyroid nodules of a 52-year-old woman was predicted with the combined model. A 52-year-old woman with a 17×12×18 mm thyroid nodule in the lower pole of the left lobe. Conventional ultrasound showed a solid hypoechoic irregular nodule with wider than taller, blurred irregular margins and suspicious microcalcifications, which were classified as C-TIRADS 4C. SWE measurement showed that *E*_max_ was 51.0 kPa. The predictive value calculated by the model was 0.326 (>0.226), which might be considered a Bethesda I nodule. The FNA result was Bethesda I.

When ultrasound features, C-TIRADS category, and *E*_max_ were combined into the analysis, the independent predictors for Bethesda I thyroid nodules changed to nodule size, C-TIRADS category, and *E*_max_. Based on this, we constructed a combined prediction model for Bethesda I thyroid nodules (**Figures 3**, **4**). The influence of age on Bethesda I thyroid nodule prediction was reduced by introducing a stiffness property of the nodule. Age was no longer an independent predictor, indicating that the combined prediction model focused more on the characteristics of the nodule itself. Compared with using independent predictors alone, the AUC of the model was 0.769, and the sensitivity was significantly improved. The prediction model established in this study is simple and convenient, which could assist clinical practice to quickly predict before the first FNA. Based on the prediction results, the optimal FNA strategy could be selected to improve the diagnosis rate of the first FNA, avoid the physical and mental trauma caused by repeated FNA, and reduce the economic cost to the patient’s family and society.

With the development of artificial intelligence (AI) technology, many scholars are exploring the use of AI technology to improve the ability of ultrasound to distinguish benign and malignant thyroid nodules and even predict the pattern of cervical lymph node metastasis (CLNM), and the research results are encouraging (2, 33–35). The deep learning AI model (ThyNet) developed by Peng, S. et al. can assist radiologists in improving the diagnostic accuracy (0.837 vs. 0.875), effectively reducing the rate of FNA (61.9% vs. 35.2%) and the rate of missed diagnosis of malignant tumors (18.9% vs. 17.0%). ThyGPT, developed by introducing ChatGPT, could effectively communicate with doctors through human–computer interaction and improve the diagnostic efficiency (35). Yao, J. et al. further identified the categories of Bethesda IV thyroid nodules through AI technology and obtained an AUC of 0.90–0.95 (2). If, based on our prediction of Bethesda I thyroid nodules, we could further predict the pathological results of nodules through AI, the FNA rate could be further reduced and the diagnostic process of thyroid nodules could be optimized.

The limitations of this study are as follows: First, this study was a single-center retrospective study that included only patients who underwent FNA and excluded nodules that did not meet the recommended criteria for FNA, leading to potential selection bias. Second, FNA procedures were performed by multiple operators, introducing inherent individual variations. Third, surgical pathological results were lacking for most cases in this study, preventing further determination of the malignant rate among Bethesda I thyroid nodules in this cohort. Fourth, this study did not compare interobserver and intraobserver variability. Finally, external validation to assess the model’s validity has not been conducted yet and should be considered in future studies.

## 5 Conclusion

In conclusion, it was found that the maximum diameter of thyroid nodules in the range of 15–20 mm, C-TIRADS category <4C, and *E*_max_ values of SWE <52.5 kPa were independent predictors for Bethesda I thyroid nodules by multivariate logistic regression analysis. We developed a combined predictive model to predict Bethesda I thyroid nodules, which provided a convenient and useful method for clinicians to predict Bethesda I thyroid nodules in advance. This will help to optimize the FNA strategy, improve the diagnosis rate of the first FNA, avoid the secondary trauma caused by repeated puncture, and reduce the time and economic cost (1, 22, 36).

## Data Availability

The data that support the findings of this study are available from the first author, upon reasonable request. Requests to access the datasets should be directed to AW, weian1976@163.com.
