# Supplementary material for: A model based on C-TIRADS combined with SWE for predicting Bethesda I thyroid nodules
Source: Front Oncol. 2024 Aug 30;14:1421088. doi: 10.3389/fonc.2024.1421088 (PMC11393783; doi:10.3389/fonc.2024.1421088)
Supplement: Supplementary file 1 [file Table1.doc]

**A model based on C-TIRADS combined with SWE for predicting Bethesda I Thyroid nodules**

An Wei^1,2^, Yu-Long Tang^3^, Shi-Chu Tang^4^, Xian-Ya Zhang^5^, Jia-Yu Ren^5^, Long Shi^6^, Xin-Wu Cui^5*^, Chao-Xue Zhang^1*^

*** Correspondence:** Corresponding Author:

Xin-Wu Cui^*^

[cuixinwu@live.cn](mailto:cuixinwu@live.cn)

Chao-Xue Zhang^*^

[zcxay@163.com](mailto:zcxay@163.com)

^†^These authors have contributed equally to this work and share corresponding authorship

C-TIRADS scoring criteria.

| **C-TIRADS Category** | | **Value of score** | **Rate of malignancy** | **Characteristics score** |
| --- | --- | --- | --- | --- |
| 1 | Absence of nodules |  |  | **-1** （Minus one score in the presence of the following characteristics）^#^ |
| 2 | Benign | -1 | 0 | Punctate hyperechoic (comet tail artifact) |
| 3 | May be benign | 0 | ＜2 | **+1** （Plus one score in the presence of the following characteristics） |
| 4A | Low suspicion | 1 | 2-10 | a、nonparallel orientation (taller-than-wide)  b、Solid  c、Very hypoechoic  d、Punctate hyperechogenicity (suspicious microcalcification)  e、Blurred/irregular margins or extrathyroidal extension |
| 4B | Intermediate suspicion | 2 | 10-50 |
| 4C | High suspicion | 3-4 | 50-90 |
| 5 | High risk of malignancy | 5 | ＞90 |
| 6 | Pathology confirmed malignancy |  |  |  |

Note: **P*-value＜0.05 was considered statistically significant.

^#^ Comet tail artifacts were scored only in the absence of suspicious microcalcifications.
